# Systemic deterrence of aphid probing and feeding by novel *β*-damascone analogues

**DOI:** 10.1007/s10340-014-0635-x

**Published:** 2014-11-25

**Authors:** Beata Gabryś, Katarzyna Dancewicz, Anna Gliszczyńska, Bożena Kordan, Czesław Wawrzeńczyk

**Affiliations:** 1 Department of Botany and Ecology, University of Zielona Góra, Szafrana 1, 65-516 Zielona Góra, Poland; 2 Department of Chemistry, Wrocław University of Environmental and Life Sciences, Wrocław, Poland; 3 Department of Phytopathology and Entomology, University of Warmia and Mazury, Olsztyn, Poland

**Keywords:** *β*-Damascone, Anti-feedants, Aphids, Probing behaviour

## Abstract

*β*-Damascone appeared a weak attractant close to not active to *Myzus persicae*, but modifications of its structure caused the avoidance of treated leaves by aphids during settling and reluctance to probe in simple choice- and no-choice experiments in previous studies. Here, the electrical penetration graph (EPG) technique, which allows monitoring of aphid probing within plant tissues, was applied to explore the biological background and localisation in plant tissues of the deterrent activities of *β*-damascone and its analogues. Activity of *β*-damascone and *β*-damascone-derived compounds depended on their substituents, which was manifested in the variation in the potency of the behavioural effect and differences in aphid probing phases that were affected. *β*-Damascone appeared a behaviourally inactive compound. The moderately active *β*-damascone ester affected aphid activities only during the phloem phase. The highly active deterrents—dihydro-*β*-damascol, *β*-damascone acetate, *δ*-bromo-*γ*-lactone, and unsaturated *γ*-lactone—affected pre-phloem and phloem aphid probing activities. The most effective structural modification that evoked the strongest negative response from *M. persicae* was the transformation of *β*-damascone into *δ*-bromo-*γ*-lactone. The behavioural effect of this transformation was demonstrated in frequent interruption of probing in peripheral tissues, which caused repeated failures in finding sieve elements, and reduction in the ingestion time during the phloem phase in favour of watery salivation. The inhibition of aphid probing at both the pre-phloem and phloem levels reveals the passage of the compounds studied through the plant surface and their distribution within plant tissues in a systemic way, which may reduce the risk of the transmission of non-persistent and persistent viruses.

## Key Message

Plant-derived chemicals are considered as alternatives to conventional neurotoxic pesticides. Modifications of natural molecules may enhance their deterrent effects, which was also the result of the present study. Depending on the substituents, behavioural effects of *β*-damascone-derived compounds varied in potency and stability. Aphid probing was impeded at the pre-ingestive (pre-phloem) and/or ingestive (phloem) phases, which revealed the passage of compounds through the plant surface and distribution within tissues in a systemic way. Inhibition of aphid probing may reduce the risk of non-persistent and persistent virus transmission.

## Introduction

Aphids (Homoptera: Aphididae) are responsible for at least 2 % of all losses in the annual world crops caused by insect feeding (Wellings et al. 1989). Additionally to the removal of assimilates from plant phloem-transporting vessels, aphids transfer virus diseases from infected to healthy plants. This activity, due to the specific piercing-sucking mode of feeding, is very efficient: it is estimated that more than 50 % of all insect-borne plant viruses are spread by aphids and it is believed that the indirect damage caused by aphids due to virus transmission exceeds their direct impact on crops (Katis et al. 2007; Brault et al. 2010). The peach potato aphid *Myzus persicae* (Sulz.) alone can transmit over 100 plant viruses among plants of over 40 families (Blackman and Eastop 1985). At the same time, *M. persicae* is one of 20 aphid species that developed clones resistant to one or more insecticides, and the resistance mechanisms are the most frequent and diverse (Dedryver et al. 2010). Typical probing activities known in aphids consist of two basic phases: the pre-ingestive pathway phase (intercellular within-plant stylet penetration including the uptake of small samples of contents from cells adjacent to the stylet route) and ingestive phase (active or passive uptake of xylem or phloem sap, respectively) (Pettersson et al. 2007). During the brief intracellular probes in the epidermis and parenchyma (mesophyll in leaves) that precede feeding in phloem vessels, aphids may transmit non-persistent and semi-persistent viruses, and when aphid stylets reach sieve elements, persistent viruses may be vectored (Prado and Tjallingii 1994; Tjallingii et al. 2010). The elimination or at least reduction of penetration of plant tissues by aphids may save plants from pathogen infection. Therefore, the disruption of the host plant selection strategy by interference in the fixed scheme of aphid activities through the application of various behaviour-modifying chemicals is one of the promising approaches that may result in a decline in aphid infestation (Pickett 1991).

Considering the high biological activity of lower terpenoids, several attempts have been made to apply these compounds or their analogues as alternatives to conventional neurotoxic chemicals in pest insect control. In our previous studies (e.g. Gabryś et al. 2005), we found that lower terpenoids of plant origin can seriously affect aphid behaviour and prevent them from feeding and settling. Citral and linalool had repellent activity, manifested in a significant decrease in time spent on leaves, a decrease in the total and mean duration of penetration, and a reduced number of probes as compared to control. Citral, linalool, *S*-limonene, *α*-ionone, and camphene were feeding deterrents that caused a reduction in the total and mean probing time by aphids and their settling on the leaves. Moreover, there was a difference in activity between the isomers of a given compound: *α*-ionone was more active than *β*-ionone, *R*-pulegone was more active than *S*-pulegone, and *S*-limonene was more active than R-limonene (Halarewicz-Pacan et al. 2003; Gabryś et al. 2005; Dancewicz et al. 2008). However, from the practical point of view, the use of plant-derived anti-feedants on a large scale is not economical, so the synthetic analogues of natural compounds, often with modified structures, are more accessible for application. For example, epoxyketones obtained from (+)-dihydrocarvone and hydroxylactones derived from (+)-nootkatone appeared to be strong deterrents to *M. persicae*; α-methylenelactones derived from (+)-3-carene and (±)-camphene were strong settling deterrents to the pea aphid *Acyrthosiphon pisum* Harris (Dancewicz et al. 2006, 2011, 2012). Moreover, while natural terpenoid piperitone appeared a rather neutral or weak deterrent to aphids, piperitone-derived saturated and halogenated lactones showed strong deterrent activities at ingestive levels against *M. persicae* (Grudniewska et al. 2011, 2013).

*β*-Damascone was discovered in the 1960s during a quest to identify the characteristic smell of Bulgarian rose oil (Demole et al. 1970). Since that time it has been widely used in perfume compositions. It has also received certain attention as a potential cancer chemopreventive and a mosquito and muscoid insecticide (Askham et al. 2005; Butler 2006; Gerhauser et al. 2009; Kaufman et al. 2011). Apart from that, information on the effect of *β*-damascone on arthropods and their behaviour is very scarce. In our previous study, using simple choice- and no-choice experiments involving freely moving aphids, we found that the natural terpenoid *β*-damascone was a very weak attractant close to not active to *M. persicae* (Gliszczyńska et al. 2014). However, chemical modifications of the *β*-damascone molecule side chain (i.e. reduction of the double bond and carbonyl group, Claisen-Johnson rearrangement, reaction of hydrolysis) followed by halolactonisation and dehydrohalogenation resulted in a change in the biological activity. Certain *β*-damascone-derived compounds induced significant changes in aphid behaviour, such as non-preference of treated leaves in the choice-test on aphid settling and/or the reduction of the total and mean probing time during initial contact with treated leaves in the no-choice 15-min behavioural study. In the cases of dihydro-*β*-damascol, *β*-damascone ester, and *δ*-bromo-*γ*-lactone, the effects occurred immediately after exposure, which suggested partial pre-ingestive or ingestive activity. The effects of *δ*-bromo-*γ*-lactone and *β*-damascone ester were durable, as the activity was also expressed during the 24-h experiment on aphid settling and the deterrent effect increased in potency over time. The unsaturated *γ*-lactone and tricyclic *δ*-lactone only evoked delayed negative responses of aphids, and we hypothesised that the post-ingestive activity of the compounds might have been the reason (Gliszczyńska et al. 2014). An ideal anti-feedant is expected to inhibit insect feeding through the contact with its taste receptors. In this respect aphids differ from chewing insects because their mouthparts lack external contact chemoreceptors (Wensler and Filshie 1969) and the ingestion of sap from sieve elements is crucial for the acceptance of the host plant (Harrewijn 1990). However, during stylet penetration towards the vascular tissue, aphids take up small sap samples from parenchymal cells for gustatory purposes, which helps the recognition of the host plants (Martin et al. 1997; Gabryś and Tjallingii 2002). This special method of probing and feeding makes aphids good tools for studying tissular localisation of deterrent factors (Gabryś and Pawluk 1999). Thus, it is possible to evaluate the capability of artificially applied substances to enter parenchymal and vascular tissues through the plant surface. An anti-feedant, especially one targeted at aphids, should possess these qualities (Chapman 1974).

The promising results of our previous experiments (Gliszczyńska et al. 2014) encouraged us to further explore the biological background of the activity of *β*-damascone and its synthetic derivatives using the electrical penetration graph (EPG) technique, which allows the monitoring of aphid probing within plant tissues, which is not possible using simple choice- or no-choice tests. In particular, we were interested in testing the hypothesis that the activities of *β*-damascone-derived dihydro-*β*-damascol, *δ*-bromo-*γ*-lactone, and *β*-damascone ester, which were expressed as immediate negative responses to the treated substrate, were linked to the pathway, i.e. the pre-ingestive (=pre-phloem) phase in aphid probing, thus affecting taste receptors in the epipharyngeal organ. Accordingly, we tested the hypothesis that the activity of compounds responsible for the prolonged (*δ*-bromo-*γ*-lactone and *β*-damascone ester) or delayed (unsaturated *γ*-lactone and tricyclic *δ*-lactone) responses of aphids may be linked to the ingestive phase in aphid probing, that is, to the phase after phloem sap has been consumed by aphids, thus affecting the mechanisms of feeding rather than chemoreception. For comparison, we also examined the behavioural effects of the remaining *β*-damascone derivatives (dihydro-*β*-damascone, *β*-damascone-acetate, *γ*-bromo-*δ*-lactone *δ*-chloro-*γ*-lactone, and *γ*-chloro-*δ*-lactone) that did not cause any changes in aphid behaviour in the crude choice- and no-choice tests in the previous study (Gliszczyńska et al. 2014). Consequently, the present work comprises the results of the in vivo electrophysiological EPG studies on the probing and feeding behaviour of *M. persicae* exposed to *β*-damascone and its synthetic analogues. Following electrophysiological experiments, we attempted to link the anti-feedant activity with specific changes in the *β*-damascone molecule, especially the halolactonisation. The physiological and behavioural outcomes of this study are discussed in respect to epidemiological consequences relating to virus transmission. The localisation of deterrent activities of *β*-damascone-derived analogues in plant tissues is considered as well.

## Materials and methods

### Chemicals

**Fig. 1:**
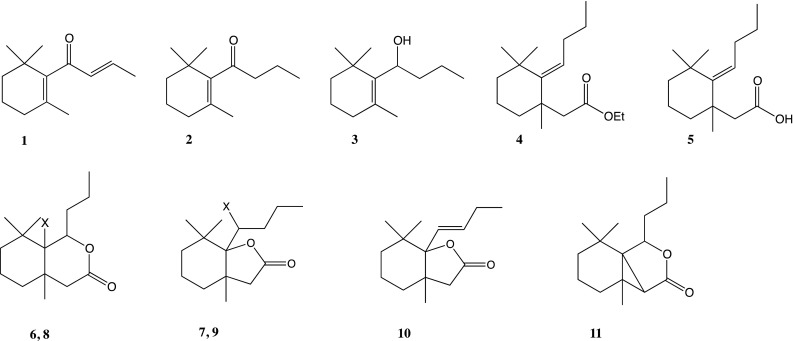
Chemical structures of *β*-damascone (**1**) and its studied analogues (**2**–**11**)

Compounds studied for the probing and feeding behaviour of *M. persicae* are illustrated in Fig. 1. *β*-Damascone (**1**) was purchased from Sigma-Aldrich. All ten chemical derivatives (**2**–**11**) of *β*-damascone (**1**) were obtained by chemical synthesis as described by Gliszczyńska et al. (2014). The first step of synthesis was the reduction of *β*-damascone (**1**) with LiAlH_4_ to ketone-dihydro-*β*-damascone (**2**), which was subsequently transformed into corresponding allylic alcohol-dihydro-*β*-damascol (**3**). The Claisen-Johnson rearrangement (orthoacetate modification) of alcohol (**3**) was the key step of the described synthesis. The product of this rearrangement, *γ*, *δ*-unsaturated ethyl ester—ethyl 2-(2-butylidene-1,3,3-trimethylcyclohexyl)-acetate **(4)**, was next hydrolyzed (KOH, EtOH) to 2-(2-butylidene-1,3,3-trimethylcyclohexyl) acetic acid (**5)**. Product **(5)** was transformed into *δ*-halo-*γ*-lactones: 7a-(1-bromobutyl)-3a,7,7-trimethylhexahydrobenzofuran-2-one **(6)**, 7a-(1-chlorobutyl)-3a,7,7-trimethylhexahydrobenzofuran-2-one **(8)** and *γ*-halo-δ-lactones: 7a-bromo-3a,7,7-trimethyl-8-propyloctahydroisochromen-3-one **(7)** and 7a-chloro-3a,7,7-trimethyl-8-propyloctahydroizochromen-2-one **(9)** in the bromo- and chlorolactonisation process under basic conditions (NBS/NCS, THF). The lactones 7a-((E)-but-1-enyl)-3a,7,7-trimethylhexahydrobenzofuran-2-one **(10)** and 3a,7,7-trimethyl-8-propylhexahydro,cyclopropa[1,2]benzofuran-2(3H)-one **(11)** were the products of the dehydrohalogenation reaction of the respective *δ*-halo-*γ*-lactones **(6)**, **(8)** and *γ*-halo-*δ*-lactone **(7)**, and **(9)** with 1,8-diazabicyclo[5.4.0]undec-7-ene (DBU).

### Bioassays

#### Insect and plant cultures and application of compounds

Aphids (*Myzus persicae*) (kept as a multiclonal colony) and plants (Chinese cabbage *Brassica pekinensis*) were reared in a laboratory at 20 °C, 65 % r.h., and 16:8 (L/D) photoperiod. One- to 7-day-old apterous females of *M. persicae* and 3-week-old plants with 4–5 fully developed leaves were used for experiments. All experiments were carried out under the same conditions of temperature, relative humidity, and photoperiod. The bioassays were started at 10–11 a.m. The compounds were applied to one leaf of a plant by immersing it in 0.1 % ethanolic solution of a given compound for 30 s. Control leaves of similar size were immersed in 70 % ethanol, which was used as a solvent for *β*-damascone and its studied derivatives. Treated and control leaves were allowed to dry for 1 h before the start of the experiment to permit the evaporation of the solvent.

#### Behavioural responses of aphids during probing and feeding

The anti-feedant effect of *β*-damascone and its structural analogues was monitored using the technique of electronic registration of aphid stylet penetration in plant tissues referred to as EPG. This technique is commonly applied in Hemiptera-plant relationship studies (Golawska and Lukasik 2012; Golawska et al. 2014). In this experimental setup, the aphid and plant are made parts of an electric circuit, which is completed when the aphid inserts its stylets into the plant. Weak voltage is supplied in the circuit, and all changing electric properties are recorded as EPG waveforms that can be correlated with aphid activities and stylet position in plant tissues (Tjallingii 1994). The values of parameters derived from EPG recordings, e.g. the duration of probing, duration of phloem sap ingestion, number of probes, etc., reflect the level of suitability of a food source for aphids (Mayoral et al. 1996). After the attachment of the golden wire electrode, aphids were starved for 1 h prior to the experiment. Probing behaviour of 16 apterous females per substance studied was continuously monitored for 8 h with an eight-channel DC EPG recording device. Each aphid was given access to a freshly prepared leaf. Signals were saved on the computer and analysed using PROBE 3.1 software provided by W.F. Tjallingii (EPG-Systems; Dillenburg 12, 6703 CJ Wageningen, The Netherlands). The following EPG patterns were distinguished: np (non-penetration—aphid stylets outside the plant),* C* (pathway phase—penetration of non-phloem tissues, including *F* = derailed stylet activities and *G* = xylem sap ingestion), *E1* (salivation into the sieve elements), and *E2* (ingestion of phloem sap). The *E1*/*E2* transition patterns were included in *E2*. A number of sequential (i.e. describing the sequence of events during the recording) and non-sequential (i.e. referring to the frequency and total and average duration of patterns) parameters were calculated (van Helden and Tjallingii 1993) and analysed in a configuration related to the activities in peripheral and vascular tissues.

### Statistical analysis

The Mann-Whitney *U* test was applied on the non-transformed data. All calculations were performed using the STATISTICA 6.1 package (StatSoft, Tulsa, OK, USA).

## Results

The EPG recording revealed all kinds of aphid activities related to plant penetration: non-probing, pathway phase ‘*C*’ including the unidentified (‘derailed’) stylet movements ‘*F*', phloem watery salivation and sap ingestion ‘*E1*' and ‘*E2*', respectively, and xylem sap uptake ‘*G*'. ‘*F*' and ‘*G*' activities occurred sporadically irrespective of the treatment.

**Fig. 2:**
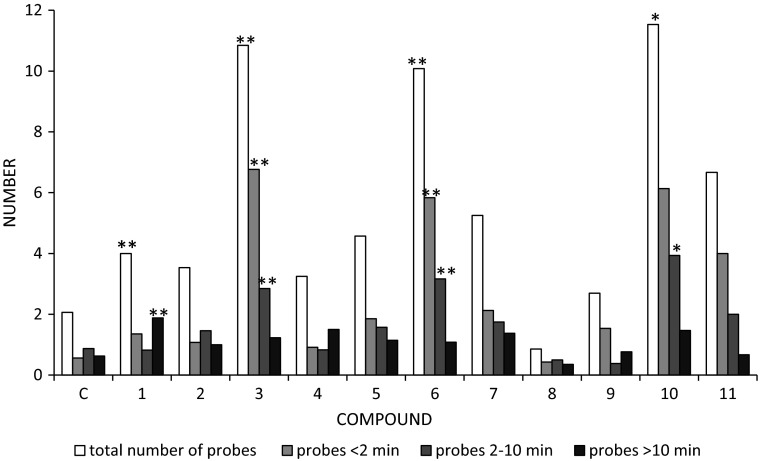
Number and duration of probes preceding the first phloem phase in *Myzus persicae* exposed to *β*-damascone (**1**) and its analogues (**2**–**11**). *Asterisks* indicate significant differences in relation to control (C) at *P* < 0.01 (*) or *P* < 0.001 (**) (Mann-Whitney *U* test)

**Fig. 3:**
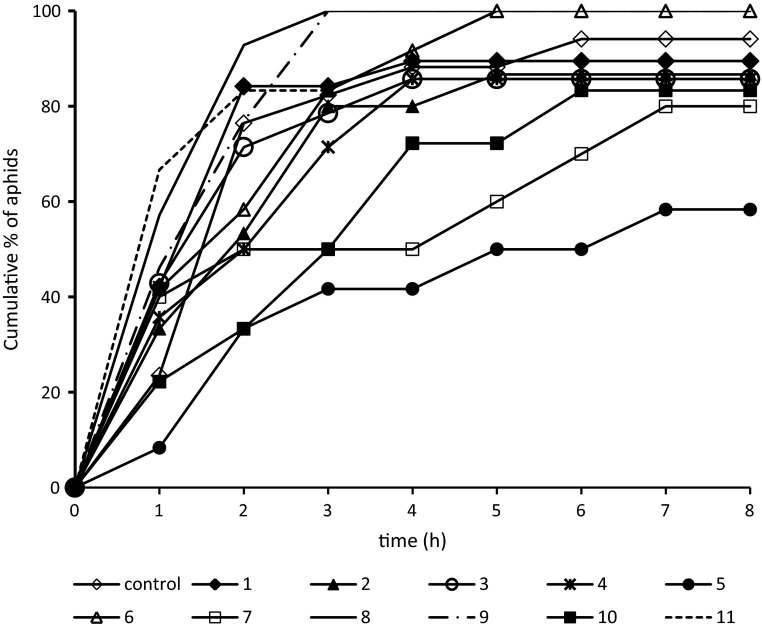
Trends in the proportion of aphids that made contact with sieve elements during 8-h access to plants after exposure to *β*-damascone (**1**) and its analogues (**2**–**11**)

The typical behaviour of *M. persicae* on control untreated plants consisted mainly of activities associated with pathway and phloem phases: 36 and 57 % of the experimental time, respectively. Aphids rarely withdrew their stylets from plant tissues (six times during the 8-h EPG recording on average) and the pauses between probes were short, 5 min on average. The individual probes were relatively long (1.2 h average duration) and nearly 60 % of them were successful, i.e. during those probes aphids reached phloem vessels (Table 1). The number of failed probes before finding phloem was relatively low: two, usually short epidermal probes per aphid (Fig. 2), and the total time of non-probing preceding the first contact with phloem vessels was 7 min on average (Table 2). The first probe was relatively long (4.5 h on average) and it typically comprised a sustained sap ingestion period (2.8 h long on average in more than 50 % of aphids) (Tables 1, 2). Nearly 80 % of aphids reached phloem vessels in the second hour after having access to the plants and nearly 90 % of aphids showed sustained ingestion by the end of the experiment (eight phloem phases per aphid on average) (Table 2; Fig. 3). The phloem phase consisted mainly of passive sap ingestion activity; the contribution of* E1* salivation to the phloem phase was 6 % (Table 1).

**Table 1:** General aspects of the probing behaviour of *Myzus persicae* after the application of *β*-damascone (**1**) and its analogues (**2–11**)

Compound	Total duration of non-probing np (min)	Total duration of pathway C + F + G (min)	Proportion of phloem phase in total probing (%)	Proportion of salivation in phloem phase (%)	Number of probes (#)	Duration of 1st probe (min)	Proportion of probes with phloem phase (%)
C	33.8 ± 18.4	172.0 ± 31.7	57.3 ± 8.0	6.1 ± 2.4	6.4 ± 2.0	249.1 ± 54.4a	57.1 ± 9.7
1	76.1 ± 21.06a	203.6 ± 24.50a	43.4 ± 7.2	6.7 ± 3.2	13.6 ± 2.8a	23.9 ± 4.8a	31.6 ± 6.9
2	77.9 ± 23.0a	205.7 ± 29.0a	44.1 ± 8.7	1.9 ± 0.7	10.4 ± 1.9	105.2 ± 43.6a	33.9 ± 9.5
3	116.9 ± 111.4a	175.6 ± 126.6a	48.9 ± 38.7	41.9 ± 38.4ab	27.2 ± 5.4ab	4.6 ± 8.3ab	11.3 ± 2.3a
4	143.7 ± 35.1a	213.9 ± 25.1a	31.2 ± 6.0	6.2 ± 3.0	12.5 ± 2.8	102.6 ± 32.0b	31.0 ± 10.3
5	221.5 ± 45.2ab	202.6 ± 34.9ab	16.4 ± 6.1ab	4.4 ± 2.2	15.0 ± 2.8a	55.6 ± 25.1a	14.8 ± 8.1a
6	70.6 ± 95.6a	187.4 ± 108.0a	47.2 ± 30.4	17.9 ± 26.7ab	21.1 ± 4.6a	3.4 ± 5.2ab	23.0 ± 4.1a
7	113.2 ± 32.9a	203.0 ± 41.4a	39.0 ± 11.8	4.9 ± 2.7	10.6 ± 3.4	19.0 ± 7.4a	31.4 ± 12.1
8	19.2 ± 15.0b	270.6 ± 37.0b	40.2 ± 8.0	5.9 ± 5.3	6.1 ± 0.8	232.9 ± 59.4b	21.5 ± 3.0
9	16.2 ± 7.7b	234.0 ± 30.5b	48.8 ± 7.0	0.6 ± 0.2	11.1 ± 3.4	53.3 ± 16.0a	31.4 ± 6.8
10	165.8 ± 31.0ab	201.6 ± 28.7ab	29.17.0	4.9 ± 1.6	31.3 ± 5.8ab	18.9 ± 8.8a	22.0 ± 8.5a
11	28.3 ± 17.3b	111.7 ± 23.4b	74.4 ± 5.4b	0.2 ± 0.1b	10.9 ± 4.3	129.3 ± 61.1	43.1 ± 12.4

Values are mean ± SE. Values followed by a letter within each column show significant differences in relation to control (letter ‘a’) and damascone (letter ‘b’) at *P* < 0.05 (Mann-Whitney *U* test)

**Table 2:** Phloem sap ingestion-related activities during probing by *Myzus persicae* after the application of *β*-damascone (**1**) and its analogues (**2–11**)

Compound	Proportion of aphids with phloem phase (%)	Proportion of aphids with sustained* E2* (%)	Proportion of aphids with sustained* E2* in 1st probe (%)	Total duration of non-probing before 1st E (min)	Number of phloem phases (#)	Duration of 1st phloem phase E (min)
C	94.1 ± 5.9	88.2 ± 8.1	52.9 ± 12.5	7.2 ± 3.5	7.8 ± 2.2	160.2 ± 47.6
1	89.5 ± 7.2	84.2 ± 8.6	15.8 ± 8.6a	10.4 ± 3.1	6.7 ± 1.3	0.5 ± 0.1a
2	86.7 ± 9.1	80.0 ± 10.7	20.0 ± 10.7a	57.4 ± 40.7	5.5 ± 1.5	139.7 ± 50.1b
3	92.9 ± 26.7	71.4 ± 46.9	7.1 ± 26.7a	30.6 ± 43.1a	14.6 ± 15.9	21.6 ± 52.1a
4	85.7 ± 9.7	78.6 ± 11.4	28.6 ± 12.5	13.9 ± 4.3a	6.5 ± 1.3	81.9 ± 33.4b
5	58.3 ± 14.9	50.0 ± 15.1	25.0 ± 13.1	93.1 ± 46.5	2.4 ± 0.8ab	24.1 ± 15.1b
6	100.0 ± 0.0	83.3 ± 38.9	0.0 ± 0.0a	23.4 ± 18.9ab	14.3 ± 11.4ab	0.7 ± 0.7a
7	80.0 ± 13.3	80.0 ± 13.3	60.0 ± 16.3	57.3 ± 32.8	2.4 ± 0.7	138.1 ± 66.5b
8	100.0 ± 0.0	100.0 ± 0.0	50.0 ± 13.9b	9.0 ± 6.2b	8.1 ± 1.0	81.1 ± 33.4b
9	100.0 ± 0.0	100.0 ± 0.0	30.8 ± 13.3	12.1 ± 7.8	7.6 ± 1.6	94.7 ± 39.2b
10	83.3 ± 9.0	66.7 ± 11.4	44.4 ± 12.1	70.3 ± 21.9ab	3.7 ± 0.6	41.5 ± 21.8b
11	100.0 ± 0.0	100.0 ± 0.0	100.0 ± 0.0ab	11.9 ± 2.9	1.5 ± 0.3ab	274.4 ± 40.7b

Values are mean ± SE. Values followed by a letter within each column show significant differences in relation to control (letter ‘a’) and damascone (letter ‘b’) at* P* < 0.05 (Mann-Whitney *U* test)

Aphids on *β*-damascone **(1)**-treated leaves showed a slight increase in the total duration of non-probing and pathway activities, but no effect on the overall duration of sap ingestion activity occurred. The proportion of phloem phase in total probing was similar, and the number of probes during the whole experimental time was higher and their duration shorter (less than 0.5 h on average) compared to aphids on control leaves (Table 1). Nevertheless, although probes before the first phloem phase were more numerous and mainly epidermis/mesophyll deep, i.e. less than 2 or 2–10 min long (Fig. 2), the time of non-probing before the first period of ingestion was comparable to that on control plants (Table 2). The proportion of aphids with sustained ingestion in the first probe was 3.3 times lower than on control plants (Table 2) but nearly 85 % of aphids reached phloem vessels in the second hour after having access to the plants and nearly 85 % of aphids finally ingested sap in a sustained way (Table 2, Fig. 3). The phloem phase consisted mainly of passive sap ingestion activity; the contribution of* E1* salivation to the phloem phase was 6 % (Table 1).

The application of *β*-damascone (**1**) analogues with various modifications in the derived structures evoked a wide spectrum of changes in aphid behaviour. All analogues except the chlorinated lactones, δ-chloro-γ-lactone (**8**) and γ-chloro-δ-lactone (**9**), and tricyclic δ-lactone (**11**) caused a slight increase in the total duration of aphid non-probing and pathway activities but had no significant effect on the overall duration of sap ingestion activity (Table 1). An increase in the duration of non-probing activities and the number of failed probes before the first period of sustained ingestion was evoked by all *β*-damascone (**1**) analogues except *β*-damascone acetate (**5**), *δ*-chloro-*γ*-lactone (**8**), *γ*-chloro-*δ*-lactone (**9**), and tricyclic *δ*-lactone (**11**). The failed probes were usually epidermis/mesophyll deep (Fig. 2). The duration of the first probe and the proportion of aphids with sustained ingestion during that probe decreased after the application of dihydro-*β*-damascone (**2**), dihydro-*β*-damascol **(3)**, *β*-damascone acetate (**5**), *δ*-bromo-*γ*-lactone (**6**), *γ*-bromo-*δ*-lactone (**7**), *γ*-chloro-*δ*-lactone (**9**), unsaturated *γ*-lactone (**10**) and dihydro-β-damascone (**2**), dihydro-β-damascol (**3**), and *δ*-bromo-*γ*-lactone (**6**), respectively (Tables 1, 2). On *δ*-bromo-*γ*-lactone (**6**)-treated leaves, no aphid showed sustained ingestion during the first probe. On dihydro-*β*-damascone **(2)** and dihydro-*β*-damascol **(3)**-treated leaves, the proportion of aphids with *E2* > 10 min in the first probe was very low: 20 and 7 %, respectively (Table 2). On *β*-damascone acetate (**5**)-treated leaves, 40 % of aphids failed to locate the phloem during the 8-h experiment, and those that did reach it made it considerably later than aphids on control or *β*-damascone (**1**)-treated leaves (Fig. 3). The duration of the first phloem phase was the shortest in aphids on *β*-damascone **(1)** and *δ*-bromo-*γ*-lactone (**6**) and the longest on tricyclic *δ*-lactone (**11**)-treated leaves. Additionally, the proportion of phloem watery salivation during the phloem phase increased significantly in aphids on dihydro-*β*-damascol **(3)** and *δ*-bromo-*γ*-lactone **(6)**-treated leaves (Tables 1-2).

## Discussion

The parameters derived from the electronic registration (EPG) describe aphid behaviour during probing and feeding and are good indicators of plant suitability or interference of probing by chemical or physical factors, including the exogenously applied chemicals, in individual plant tissues (Mayoral et al. 1996). The results of the experiments presented here illustrate three major aspects of biological activity of the compounds studied: (1) the general effects on aphid behaviour, (2) non-phloem/mesophyll-located pre-ingestive activity, and (3) phloem-located ingestive activity. General effects were manifested as a decrease in the duration of probing and sap ingestion, a decrease in the proportion of ingestion in total probing, an increase in non-probing time until the first phloem phase, and a lower proportion of aphids that made contact with phloem tissue and ingested sap in a sustained manner during the first probe. The non-phloem pre-ingestive activity was demonstrated as the interruption of probing at the mesophyll level, i.e. the high number of short (<2 min.) probes vs. long probes before reaching sieve elements, the high proportion of non-probing before the first phloem phase, the shorter duration of the first probe, and the lower proportion of aphids with sap ingestion periods during the first probe in comparison to the control. Phloem ingestive activity was expressed as a shorter duration of the first phloem phase and sap ingestion phase *E2*, a lower proportion of aphids with sustained ingestion in general and a lower proportion of aphids with sustained ingestion in the first probe in particular, and a higher proportion of salivation during the phloem phase in relation to the control. Long total probing times and long individual probes, especially those that include the phloem phase, indicate the absence of negative factors in the epidermis and/or parenchyma that would cause the withdrawal of stylets and discontinuation of the route towards sieve elements. Likewise, the frequency and duration of phloem phases may show the effect of phloem sap composition on plant acceptability. The duration of the salivation period during the phloem phase is positively correlated with plant resistance. That is, on resistant plants or non-hosts, phloem salivation may appear the major or only aphid activity in sieve elements (van Helden and Tjallingii 1993; Klinger et al. 1998; Wilkinson and Douglas 1998; Gabryś and Pawluk 1999). In contrast, on suitable host plants, the sap ingestion periods may last for many hours with no interruption (Alvarez et al. 2006; Montllor and Tjallingii 1989; Marchetti et al. 2009). Accordingly, the alteration of aphid behaviour during the pathway phase may reflect the hindrance of probing at the pre-ingestive level, the changes in behaviour during contact with phloem elements—at the ingestive one, while the refusal to settle on plants even if the feeding process has not been impeded may be a symptom of post-ingestive deterrence (Frazier and Chyb 1995; Grudniewska et al. 2011, 2013).

The analysis of the structural characteristic of *β*-damascone and its analogues in the context of the behavioural effects induced by the application of individual substances allows dividing the compounds studied into three groups in terms of activity and the significance for virus transmission: (1) not active, (2) moderately active, and (3) highly active. The compounds defined as not active affected aphid behaviour at neither the pre-phloem or phloem phases or their effect was negligible. β-Damascone **(1)**, dihydro-*β*-damascone **(2)**, *γ*-bromo-*δ*-lactone **(7)**, *δ*-chloro-*γ*-lactone **(8)**, and *γ*-chloro-*δ*-lactone **(9)** are included in this group. This conclusion is in accordance with the findings in the previous study: neither of the compounds listed had any effect on free aphid settling in the 24-h choice experiment (Gliszczyńska et al. 2014). The moderately active analogues of *β*-damascone affected aphid activities only during the phloem phase. The disturbance in sap ingestion periods might cause the decrease in direct damage due to the removal of assimilates from the sieve elements. At the same time, the limitation of transmission of circulative persistent viruses is possible. This group of deterrents comprises *β*-damascone ester (**4**), which significantly reduced the total sap ingestion time. Moreover, the deterrent effect was relatively durable: aphids refused to settle on plants for at least 24 h after exposure (Gliszczyńska et al. 2014). The highly active deterrents among the compounds studied were dihydro-*β*-damascol (**3**), *β*-damascone acetate (**5**), *δ*-bromo-*γ*-lactone (**6**), and unsaturated *γ*-lactone (**10**). Their addition affected pre-phloem and phloem activities. The difficulties and overall failure in finding sieve elements and the frequent interruption of probing after short periods may contribute to the limitation of transmission of non-persistent, mesophyll-related viruses as well as persistent viruses, the transmission of which requires phloem sap ingestion. However, there were differences in the potency and mechanism of expression of deterrent activity among these *β*-damascone analogues. In the case of dihydro-*β*-damascol (**3**), the first phloem phase was very short, very few aphids continued ingestion during the first phloem phase, and the contribution of watery salivation was very high. All these would indicate a high ingestive activity. However, in the choice test, no significant difference was found in the number of aphids settling on the treated and untreated leaves during the 24-h experiment (Gliszczyńska et al. 2014). Possibly, the detrimental effects of the phloem-associated deterrent components were overcome by intensive watery salivation (more than 40 % of all phloem-related activities). This type of aphid saliva contains a complex enzymatic system for the detoxification of allelochemicals (Tjallingii 2006). *β*-Damascone acetate (**5**) caused a failure in locating sieve elements in many aphids and a reduction in phloem sap ingestion time in the other ones, but the proportion of watery salivation in the phloem phase was low. Therefore, in the settling choice experiment, the deterrent effect was weak and occurred 24 h after exposure (Gliszczyńska et al. 2014). In contrast, *δ*-bromo-*γ*-lactone (**6**) may be considered a potent deterrent, probably showing a post-ingestive activity in addition to the pre- and ingestive ones. The frequently interrupted probing prior to the first contact with sieve elements and the significantly lower proportion of successful probes in relation to the control indicate the activity in peripheral tissues; the reduced duration of the phloem phase suggests the activity at the phloem level. The tethering of aphids in the EPG experiments prevents them from walking away from unsuitable food sources. Indeed, in the choice test, the freely moving aphids avoided the treated leaves from the very beginning until the end of the 24-h experiment (Gliszczyńska et al. 2014). The unsaturated *γ*-lactone (**10**) produced similar effects to those evoked by *β*-damascone acetate (**5**). However, the effect, probably post-ingestive, during the choice settling experiment was much stronger (Gliszczyńska et al. 2014). The results of the EPG experiment after the application of tricyclic *δ*-lactone (**11**) suggest that it had attractant properties: the number of probes was low, the first probe was long, and all aphids that reached phloem for the first time continued feeding in a sustained way. However, the choice experiment on free-moving aphids demonstrated that aphids avoided the treated leaves 24 h after exposure (Gliszczyńska et al. 2014). It is likely that tricyclic *δ*-lactone **(11)** has post-ingestive deterrent properties despite its probing-promoting activity during stylet penetration in plant tissues, which was shown in the present study.

Pre-ingestive inhibitors that interfere with food selection and consumption by insects are supposed to affect the response of gustatory receptors, ingestive inhibitors probably suppress salivary enzymes or the transport of food, and post-ingestive inhibitors are thought to affect physiological processes after food had been ingested (Frazier and Chyb 1995). Various mechanisms of these phenomena have been proposed: the change in the activity of receptors that signal the presence of feeding stimulants, the alteration of signal codes due to the stimulation of specialised receptors, or biogenic amine inhibition (Koul 2005). It is generally accepted that various molecular parameters such as chirality, functional groups, molecular size, and lipophilicity of the compounds are associated with the biological activity, including feeding deterrence mechanisms. Variations, such as incorporation of functional groups, epoxidation, or lactonisation, can produce radical changes in the biological activity profile. For example, the presence of the *α*-methylene-*γ*-lactone moiety is considered essential for the anti-tumour activity of sesquiterpene lactones because of its ability to alkylate biological macromolecules, which may result in deactivation of the nucleophilic centres of biological targets such as key enzymes, which control cell division, thereby inhibiting a variety of cellular functions (Chaturvedi 2011; Orofino-Kreuger et al. 2012). Unfortunately, any generalisation based on functionality and skeletal types of natural or synthetic compounds is difficult to produce in anti-tumour research (Chaturvedi 2011) as well as in relation to feeding deterrent activity (Koul 2005). As for anti-feedants, the bioactivity is highly species- and developmental stage-specific. For example, within a group of synthetic linalool-derived alkyl-substituted* γ*- and* δ*-lactones, the unsaturated lactones were the strongest anti-feedants for Colorado potato beetle *Leptinotarsa decemlineata* larvae and adults, saturated lactones with three alkyl substituents were deterrent only to its larvae, while the settling of *M. persicae* on plants was strongly deterred only by iodolactones (Gabryś et al. 2006). Camphene, which was a strong deterrent to *M. persicae*, lost the activity after the incorporation of the α-methylenelactone moiety, while the same camphene-derived α-methylenelactone was a strong deterrent to the pea aphid *Acyrthosiphon pisum* (Dancewicz et al. 2006). Therefore, to obtain crucial information about the optimal relative stereochemistry required to stimulate bioactivity, including the anti-feedant response in a given insect species, a critical examination of functional groups present in the active molecules is needed. The results of the experiments shown in the present work illustrate three major aspects of the biological activity of *β*-damascone and its analogues that depend on their substituents: (1) the variation in the potency of the behavioural effect, (2) the stability of the deterrent effects, and/or (3) a switch from attractant to deterrent properties or otherwise. The most effective structural modification that evoked the strongest negative response from *M. persicae* was the transformation of *β*-damascone into *δ*-bromo-*γ*-lactone (**6**). The behavioural effect of this transformation was manifested as frequent interruption of probing in peripheral tissues, which caused repeated failures in finding sieve elements, and the reduction in the ingestion time during the phloem phase in favour of watery salivation. The tethering of aphids, which is the major limitation of the EPG experiments, prevented them from walking off from the unsuitable food source, which was the reaction to the δ-bromo-*γ*-lactone (**6**) in the choice and no-choice experiments with freely moving individuals (Gliszczyńska et al. 2014). The inhibition of aphid probing at both the pre-phloem and phloem levels may reduce the risk of the transmission of non-persistent and persistent viruses. Additionally, the modifications of aphid probing behaviour at the pre-ingestive and ingestive phases by the *β*-damascone analogues demonstrated in the present study, as well as the comparative data from plants (Gabryś and Pawluk 1999; Douglas 2003), reveal the passage of the compounds studied through the plant surface and the distribution within plant tissues in a systemic way.

## Author contributions

BG: conceived the biological experiments and wrote the manuscript, KD performed the biological experiments and analysed data, AG designed and performed the chemical experiments, BK analysed the biological data, and CW conceived the chemical experiments. All authors read and approved the manuscript.
